# Serum heat inactivation diminishes ApoE-mediated uptake of D-Lin-MC3-DMA lipid nanoparticles

**DOI:** 10.3762/bjnano.16.57

**Published:** 2025-05-30

**Authors:** Demian van Straten, Luuk van de Schepop, Rowan Frunt, Pieter Vader, Raymond M Schiffelers

**Affiliations:** 1 CDL Research, University Medical Center Utrecht, Utrecht, The Netherlands

**Keywords:** apolipoprotein E, fetal calf serum, heat inactivation, lipid nanoparticle, protein corona

## Abstract

Nanoparticles play a crucial role in drug delivery research. The protein corona that develops on the surface of nanoparticles after administration has garnered substantial attention due to the significant effects it has on their performance. Lipid nanoparticles (LNPs) depend on protein corona formation to mediate their targeting. Such protein–nanoparticle interactions are often initially studied using in vitro cellular models aiming to eventually understand biodistribution and cargo delivery efficiency of the LNPs in vivo. For in vitro cell culture, fetal calf serum (FCS) is supplemented to culture media to provide nutrients and promote cell viability and growth. Heat inactivation of FCS is often performed to prevent complement system activation. However, the effect of this process on protein corona formation and, in turn, LNP functionality is unclear. Here, we investigated the effects of serum heat inactivation on protein corona formation on LNPs containing D-lin-MC3-DMA (MC3) or C12-200 (C12) ionizable lipids. Cellular uptake and siRNA delivery efficiency of the LNPs were determined in media containing untreated or heat-inactivated serum. Mechanistically, we found that apolipoprotein E, a protein corona component that is crucial for MC3 LNP tropism, displayed reduced stability and functionality upon heat inactivation of FCS, thereby negatively influencing uptake and cargo delivery of MC3 LNPs, but not C12 LNPs. Our results underline the importance of overlooked factors in in vitro experiments that can inadvertently affect LNP performance. These findings can help to improve protocols to study protein corona formation in vitro and prevent bias in LNP development*.*

## Introduction

Nanotechnology has gained a strong foothold in the field of drug delivery, having significant promise to overcome the inherent limitations of different classes of therapeutics, ranging from small molecule drugs, to biologicals such as proteins and nucleic acids. Nanoparticles can enhance the solubility and stability of their payload, prolong its circulation time, and improve its biodistribution to increase their safety and efficacy [1].

It is becoming increasingly clear that the biological fate and overall performance of nanoparticles are influenced by their interaction with the bioenvironment. As a nanoparticle interacts with a biological matrix upon administration, a layer of biomolecules, primarily composed of proteins, forms on its surface. This so-called protein corona significantly affects the physicochemical properties of the nanoparticle, such as size, charge and stability [2–5]. In turn, the composition of the protein corona is influenced by the physicochemical properties of the pristine nanoparticle, reviewed by [6–8], as well as the protein source of the corona [9–11]. Ultimately, the protein corona can change the uptake [12–14], biodistribution [15–18], immunological responses [19–20] and toxicity [6,21–22] of nanoparticles and its characterization should thus play a crucial part during pre-clinical nanoparticle development.

The influence of the protein corona is particularly evident for the efficacy of lipid nanoparticles used for RNA delivery. Lipid nanoparticles (LNPs) protect the encapsulated RNA from premature clearance and simultaneously facilitate the internalization of RNA molecules by target cells [23]. Several LNP encapsulated RNA-based therapeutics have achieved approval by the United States Food and Drug Administration (FDA) [24], including patisiran/Onpattro for the treatment of hereditary transthyretin amyloidosis. Onpattro LNPs are administered intravenously after which they are dependent on the adsorption of the plasma protein apolipoprotein E (ApoE) to their surface to efficiently target LDL-receptor expressing cells in the liver and deliver the siRNA cargo [25–26]. As the protein corona seems to play a pivotal role in LNP tissue distribution and delivery efficiency, it is becoming increasingly clear that the in vivo environment to which the nanoparticles will be exposed should be considered during LNP development. Nevertheless, the initial screening of nanoparticles in vitro is often restricted to experiments using tissue culture media supplemented with fetal calf serum (FCS). FCS is commonly heat treated to inactivate mycoplasma and viruses, as well as denature heat labile complement factors without affecting the nutrients needed for cell growth [27]. As some cell lines are sensitive to complement factors, the heat treatment can prevent the complement from interfering with the outcome of experiments. However, studies describing LNP development rarely report whether untreated (non-heat-inactivated (NHI)) or heat-inactivated (HI) FCS was used. Consequently, the effect of heat treatment of FCS on in vitro LNP protein corona formation and subsequent cell-nanoparticle interactions remains unclear.

Here, the effect of FCS heat inactivation on the in vitro behavior of the clinically applied and most studied D-Lin-MC3-DMA (MC3) LNPs was investigated and compared to LNPs containing a different ionizable lipid, C12-200 (C12). Moreover, mechanisms by which FCS heat inactivation could affect cellular interactions with LNPs were studied in detail.

## Materials and Methods

### Buffers, reagents, materials

Phosphate buffered saline (PBS), bovine serum albumin (BSA), thioflavin T (ThT), cholesterol and distearoylphosphatidylcholine (DSPC) were purchased at Sigma-Aldrich. D-Lin-MC3-DMA and C12-200 were obtained from GKV Bio. 1,2-Dimyristoyl-*rac*-glycero-3-methoxypolyethylene glycol-2000 (DMG-PEG(2000)), 1,2-dioleoyl-*sn*-glycero-3-phosphocholine (DOPC) and 1,2-distearoyl-*sn*-glycero-3-phosphoethanolamine-*N*-[biotinyl(polyethylene glycol)-2000] (DSPE-PEG(2000)-biotin) were obtained from Avanti. Human recombinant ApoE3 was purchased from Fitzgerald Industries. Mouse monoclonal anti-ApoE (NB110-60531; Bio-Techne), FITC labelled goat anti-mouse secondary antibody (A28175; ThermoFischer). Anti-firefly siRNA molecules were ordered as individual strands at Integrated DNA Technologies and were annealed in-house for 5 min at 97 °C. siRNA sequence: Sense: ‘5-GGA CGA GGU GCC UAA AGG AdCdG-3’ Antisense: ‘5-UCC UUU AGG CAC CUC GUC CdCdG-3’. FCS was obtained from Gibco, Biowest and Lonza.

#### Cell culture

Brain cancer cell line U87-MG (ATCC) and breast cancer cell line MDA-MB-231 (ATCC) were cultured in DMEM medium with 10% FCS (Gibco) and 1% penicillin-streptomycin (PS) (Fisher Scientific). The human dermal microvascular endothelial cell line HMEC-1 (ATCC) was cultured in MCDB-131 medium supplemented with 10% FCS (Gibco), 2 mM l-glutamine (Gibco), 10 ng/mL rhEGF (Peprotech), and 50 nM hydrocortisone (Sigma), in flasks coated with 0.1% gelatin (Merck). All cells were cultured at 37 °C with 5% CO_2_. Cell lines were transformed to express the dual luciferase system as described by Evers et al. [28]. Briefly, cell lines were transfected with a pHAGE2-PGK-FFluc-SV40-Rluc-NeoR_fusion-WPRE plasmid resulting in the stable expression of both Firefly and *Renilla* luciferase. The plasmid includes a G418-resistance gene to maintain construct expression by adding 500–1000 µg/mL G418 to the culture media. For HI and NHI FCS, a batch of FCS was split in two equal volumes of which one part was placed in a water bath at 56 °C for 30 min, while the other part was left untreated. After heat inactivation, FCS was stored at −20 °C until use.

#### LNP production

LNPs were prepared via microfluidics mixing, using a Nanoassemblr Benchtop device (Precision Nanosystems). In short, D-Lin-MC3-DMA, cholesterol, DSPC and DMG-PEG(2000) were dissolved in ethanol at a molar ratio of 50:38.5:10:1.5 and a final lipid concentration of 10 mM. For biotinylated LNPs, 1 M DSPC was substituted with 1 M DSPE-PEG(2000)-biotin. For C12 LNPs, C12-200, cholesterol, DOPC and DMG-PEG(2000) were dissolved in ethanol at a molar ratio of 50:38.5:10:1.5 and a final lipid concentration of 10 mM.

siRNA was diluted in an acetate buffer (25 mM, pH 4.0) to a final concentration of 3.3 µM for MC3 LNPs and 12.33 µM for C12 LNPs. For fluorescent LNPs, siRNA and siRNA-AF647 were mixed 2:1 v/v. For MC3 LNPs the Nanoassemblr was set to a flowrate of 4 mL/min and a flow rate ratio of 3:1 (aqueous to solvent phase) to mix the phases. For C12 LNPs, the flow rate ratio was set to 2:1 with a total flow rate of 9 mL/min. After mixing, the LNP suspensions were dialyzed against PBS overnight using a dialysis cassette (Thermo Scientific Slide-A-Lyzer, MWCO 20,000, 0.5–3 mL, dark, 4 °C). LNP stocks were stored at 4 °C in the dark.

#### LNP characterization

The hydrodynamic diameter of LNPs was determined via dynamic light scattering (DLS). LNP stocks were diluted 1:20 in PBS and analyzed at 25 °C using a ZetaSizer Nano ZS 90 (Malvern Analytical, UK). The zeta potential of LNPs was measured using a Zetasizer Nano Z (Malvern Panalytical). Before analysis, LNP stocks were diluted 1:20 in ultrapure water.

#### LNP uptake

Cells were seeded in 48-well plates the day prior to the uptake experiment at 2 × 10^4^ cells/well. The plates were pre-coated with gelatin for HMEC-1. After cells were allowed to adhere overnight, they were washed with serum free media to remove any interfering protein from the standard culture conditions. Fresh media were made containing 10% FCS (either heat-inactivated or untreated) and 1% PS. For ApoE rescue experiments, 1 µg/mL ApoE was added to the HI serum containing media. LNPs containing fluorescent siRNA were added to a final concentration of 10 nM siRNA-647 (30 nmol total siRNA). Per well, 150 µL LNP suspension (1.5 pmol total fluorescent siRNA) was added and incubated for 4 h at 37 °C. Subsequently, cells were washed with PBS and detached with trypsin (Gibco) and collected in full medium (containing 10% NHI FCS). Cells were spun down 5 min at 300*g* to remove trypsin, washed with cold PBS and then resuspended in ice cold PBS + 1% BSA (PBA) and their fluorescence was measured using a BD FACS Canto II (BD Biosciences). Cell mean fluorescence intensity (MFI) was analyzed with FACS Diva software.

#### ApoE dose response LNP uptake

A day before the experiment, HMEC-1 were seeded in a gelatin coated 48-well plate (5 × 10^4^ cells/well) and allowed to adhere overnight. The next day, serum-free medium was supplemented with ApoE and diluted with serum free medium to the desired ApoE concentrations. Part of the 1 µg/mL ApoE was heat-inactivated for 30 min at 56 °C and cooled to 37 °C afterwards. LNPs were added to the media in a 1:500 v/v ratio (11.5 nmol siRNA). Cells were washed with serum free medium before adding 150 µL ApoE media containing fluorescent LNPs. Cells were incubated for 4 h at 37 °C before collecting them and measuring the fluorescence by FACS as described in the previous section.

#### Knockdown

For knockdown experiments, dual luciferase expressing cells were seeded at 5 × 10^3^ cells/well in a 96-well plate (plates were pre-coated with gelatin for HMEC-1) and allowed to adhere overnight. Fresh media were prepared as described above. LNPs were added to the media to a final concentration of 10 nM anti-firefly luciferase siRNA and sterile filtered using a 0.45 µm PVDF filter. Per well, a total 1 pmol total siRNA was added and incubated for 48 h. Lipofectamine RNAiMAX (ThermoFisher) was used as a positive control according to manufacturer’s protocol. Afterwards, luciferase activity was determined using the Dual-Luciferase reporter system kit (Promega) according to manufacturer’s instructions. In short, after LNP uptake, the first reagent is added to the cells to lyse them and provide firefly luciferase substrate. After 10 min incubation at rt, the lysates are transferred to white lumitrac 96-well plates (Greiner) and firefly luminescence is measured using a Spectramax iD3 plate reader (Molecular Devices). Subsequently, the second reagent is added which quenches firefly luminescence and provides the *Renilla* substrate. After 10 min, *Renilla* luminescence is measured as an internal control to account for any non-specific luciferase activity knockdown such as treatment induced cell death. A ratio of both luciferase activities was used as a read out for LNP-mediated knockdown.

#### Protein thermal stability

To determine the thermal stability of ApoE, a solution of ApoE or BSA at 170 µg/mL in PBS was incubated at room temperature, 37 °C, 56 °C or 75 °C shaking at 300 rpm for 30 min. Subsequently, the solutions were transferred to a black 96 well plate (50 µL/well) after which an equal volume of 20 µM ThT in PBS was added. After 5 min at rt, the fluorescence was measured (excitation: 450 nm, emission: 505 nm) using a Spectramax iD3 plate reader (Molecular Devices).

#### Thermal stability of ApoE in human serum

Human serum was acquired from five healthy adult donors, via the Mini Donor service of the University Medical Center Utrecht (Biobank number 18-774). Approval for the study was obtained from the local ethics review board, and all participants provided written informed consent. Pooled human serum was heated for 30 min at 56 °C under agitation to acquire HI human serum. In a 96-well plate, 185 µL NHI human serum, HI human serum or PBS with ApoE (40 µg/mL) was added per well. Subsequently, 15 µL biotin-LNP suspension was added per well (100 µM final total lipid concentration). Samples were incubated 1 h at 37 °C under light agitation. Afterwards, 2 µL Dynabeads MyOne Streptavidin C1 (Fisher Scientific) prewashed with PBA were added to the samples and incubated for 1 h at 37 °C to capture LNPs. The beads were pelleted with a magnet and washed with PBA to remove unbound protein. Per well, 100 µL anti-ApoE antibody was added in PBA (1:200) and incubated for 1 h at rt. After incubation, beads were pelleted and washed with PBA. Beads were then resuspended in 100 µL goat-anti mouse antibody (Alexa fluor-488 labeled) in PBA (1:200) and incubated for 45 min in the dark at rt, under light agitation. After incubation, beads were pelleted, washed with- and resuspended in 200 µL PBA. Bead fluorescence was measured using a BD FACS Canto II. Bead MFI was analyzed with FACS Diva software.

#### Statistics

All statistical analyses were performed using Graphpad Prism 9.3.0. One-way ANOVA combined with a Tukey’s multiple comparisons test or T-tests were used. Differences were considered statistically significant at *p* < 0.05 and were annotated as ns = non-significant, * = *p* ≤ 0.05, ** = *p* ≤ 0.01, *** = *p* ≤ 0.001 and **** *p* ≤ 0.0001.

## Results and Discussion

**Figure 1:**
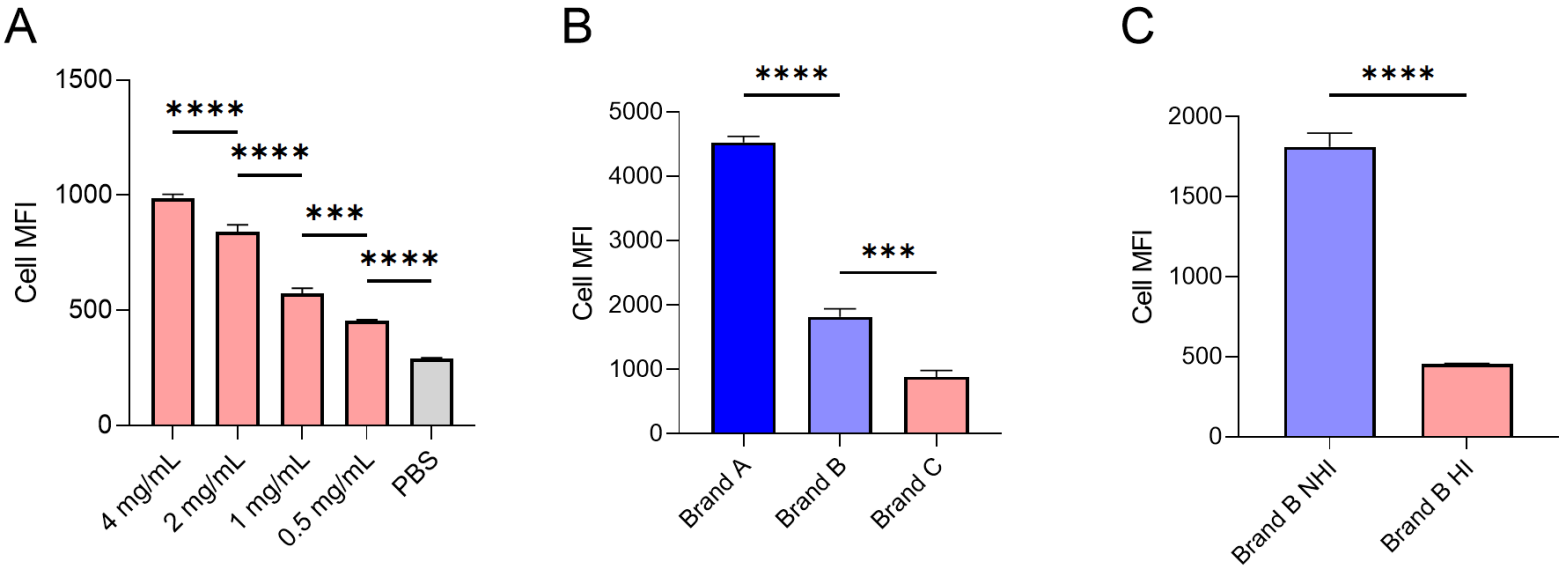
The source and treatment of FCS in culture media dictate LNP uptake by HMEC-1 cells. The mean fluorescence intensity of HMEC-1, after uptake of MC3 LNPs containing Alexa fluor 647 labelled siRNA in media supplemented with different concentrations of HI FCS (A), different brands of FCS (B) or untreated (NHI) or heat treated (HI) FCS of brand B (C) as determined by flow cytometry. The concentration of fluorescent siRNA was 1.5 pmol per 150 µL and uptake was measured after 4 h (*n* = 3). Differences were considered statistically significant at *p* < 0.05 and were annotated as ns = non-significant, * = *p* ≤ 0.05, ** = *p* ≤ 0.01, *** = *p* ≤ 0.001 and **** = *p* ≤ 0.0001.

The effect of serum heat inactivation on LNP in vitro behavior was investigated with a primary focus on biological read outs, such as LNP uptake by cells and subsequent transfection efficiency. The interest in this matter was sparked when a big variance in MC3 LNP uptake was observed between experiments, depending on the brand of FCS that was used in the cell culture media. We initially hypothesized that this difference may have been the result of differences in FCS protein concentrations. Indeed, LNP uptake by HMEC-1 increases with the concentration of (heat treated) FCS (Figure 1A). This is most likely the result of enhanced shedding of PEG-lipids from the LNP surface, which needs to occur before a protein corona can be established that allows cellular uptake of LNPs [29]. A difference in FCS protein concentration can thus introduce variations in results when performing experiments with different FCS brands or even different lots of the same supplier.

Interestingly, however, even after normalizing FCS protein concentrations, the differences in LNP uptake between different brands of FCS were significant (Figure 1B). An interesting variable between the different sources of FCS was whether or not FCS had been heat-inactivated by the manufacturer as was the case for brand C, although two brands of untreated FCS also showed a two-fold difference in uptake. Indeed, an effect of heat inactivation on LNP uptake efficiency was confirmed by comparing uptake of LNPs by cells cultured in the presence of heat-inactivated or untreated FCS taken from the same batch. Heat treatment of FCS reduced LNP uptake over threefold (Figure 1C). Together, these results suggest that, in this particular experimental setup, the type or the condition of proteins present in the FCS dictate cell interactions rather than the total amount of protein present in the media.

A similar observation was made by Simon et al. [30], who attributed a difference in cellular uptake of PEGylated polystyrene nanocarriers in untreated or heat-inactivated FCS to the denaturation of important protein corona components. Indeed, heat inactivation did not reduce the amount of protein found in the protein corona, but it did change the corona composition, thereby affecting cellular uptake of the nanoparticles. They found that some apolipoproteins unfold during heat inactivation of FCS, while other proteins remain unaffected [30].

Specifically pinpointing which proteins are significantly affected by the heat treatment is challenging due to the complexity of FCS and its derived protein corona. However, for D-lin-MC3-DMA LNPs, one corona-component, i.e., apolipoprotein E (ApoE), is particularly relevant as it has been shown to be the key component of the Onpattro protein corona that facilitates LNP binding to LDL receptors, which in turn leads to the uptake of the decorated particles by hepatocytes in the liver [25]. Therefore, we investigated the effects of heat inactivation on the stability of ApoE. We used recombinant ApoE3 for these studies, as it is the predominant form of ApoE in humans [31].

**Figure 2:**
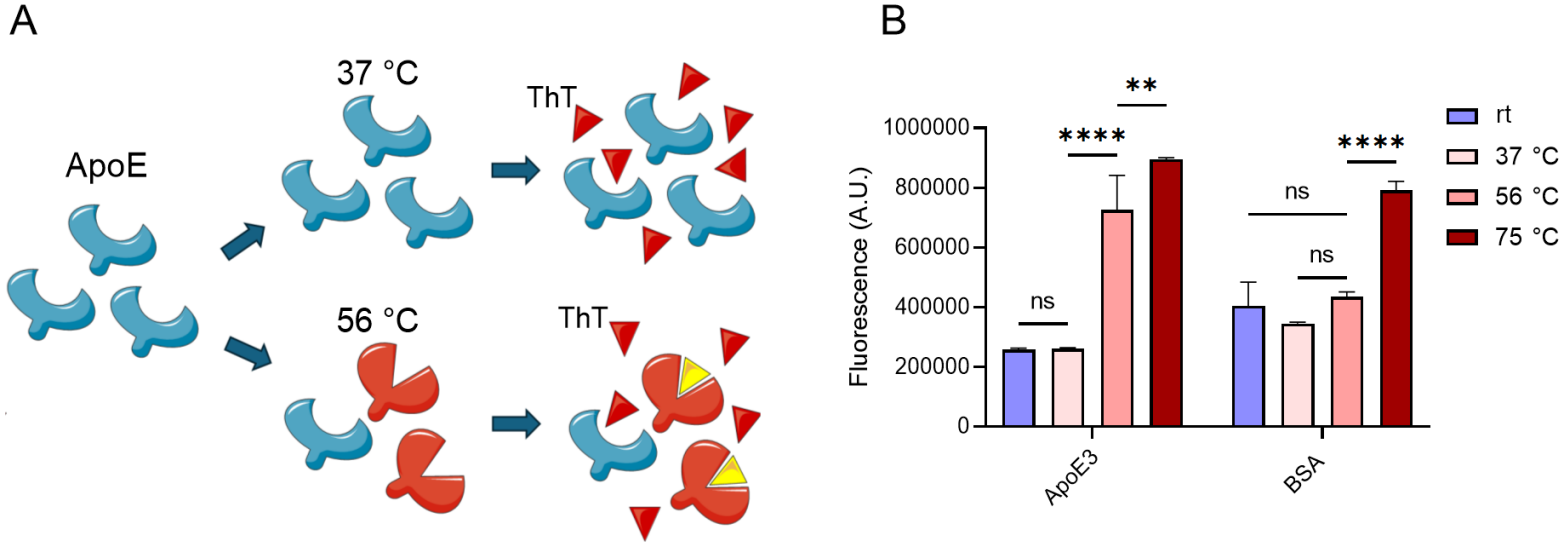
Schematic representation of the heat treatment and ThT staining of ApoE. Binding of ThT to misfolded or aggregated proteins greatly enhances its fluorescence (A). The fluorescence of solutions containing ApoE3 or BSA following ThT staining as determined by spectrophotometry, after a 30 min incubation at room temperature (rt), 37, 56 and 75 °C (*n* = 3) (B). Differences were considered statistically significant at *p* < 0.05 and were annotated as ns = non-significant, * = *p* ≤ 0.05, ** = *p* ≤ 0.01, *** = *p* ≤ 0.001 and **** = *p* ≤ 0.0001. The graphics in Figure 2A were generated with images provided by Servier Medical Art. Servier Medical Art by Servier is licensed under a Creative Commons Attribution 4.0 International License, https://creativecommons.org/licenses/by/4.0/

The most widely employed procedure for heat inactivation of FCS is 30 min incubation at 56 °C. To determine the heat stability of ApoE3, human recombinant ApoE3 was incubated for 30 min at varying temperatures between room temperature and 75 °C. Subsequently, the heat treated protein was probed with ThT, a dye that increases in fluorescence after it binds to aggregated or misfolded protein [32] (Figure 2A). Bovine serum albumin was used as a control protein as it is a major component of serum and known to be relatively heat resistant. No increase in fluorescence was seen between ApoE3 incubated at rt or at 37 °C, which is the temperature generally maintained during cell culture. However, a significant increase in fluorescence was seen after exposure of ApoE3 to 56 °C, indicating protein aggregation or denaturation. Fluorescence increased even further after incubation at 75 °C (Figure 2B). These findings are in line with published work, where thermal instability was shown for ApoE3 with an unfolding temperature of around 53 °C [33–35].

In contrast, no significant increase in fluorescence was seen for BSA at 56 °C compared to rt or 37 °C (Figure 2B), as can be expected for albumin with a reported denaturing temperature around 63 °C [36]. Indeed, after incubation at 75 °C, BSA also displayed aggregation or denaturation as shown by the increase in fluorescence of ThT. Together, this shows that during the standard FCS heat inactivating protocol, ApoE3, a key determinant of MC3 LNP uptake, is detrimentally affected while a major serum component such as BSA remains unaffected.

**Figure 3:**
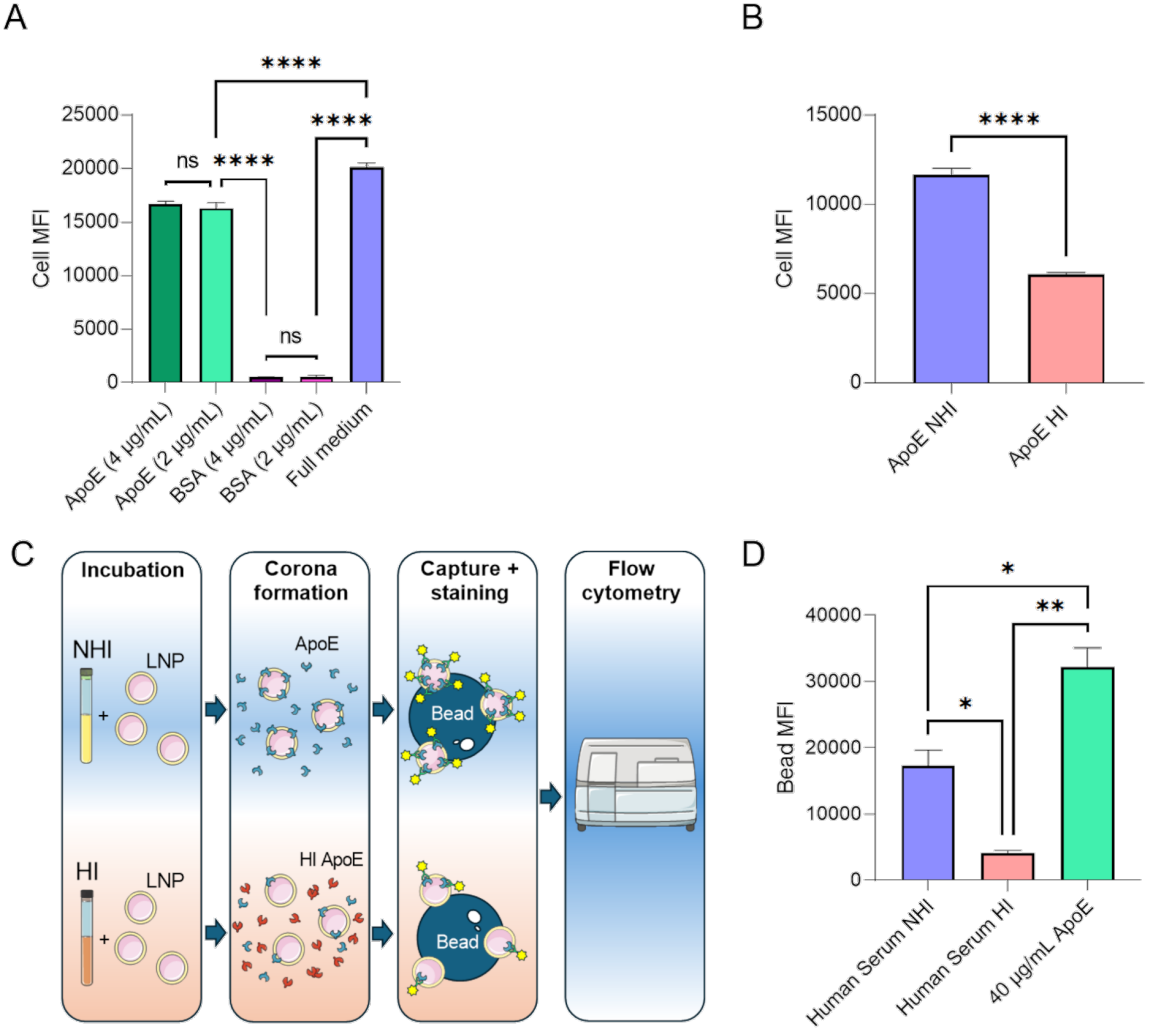
Heat-mediated aggregation/denaturation prevents ApoE from binding to LNPs and reduces LNP uptake by cells. The fluorescence of HMEC-1 after uptake of LNP containing alexa fluor 647 labelled siRNA at different concentrations of recombinant ApoE3, BSA or medium supplemented with NHI FCS (A). The concentration of fluorescent siRNA was 1.725 pmol per well and uptake was measured after 4 h (*n* = 3). The fluorescence of HMEC-1 cells after uptake of LNPs in medium supplemented with HI or NHI ApoE (B). Schematic representation of the bead capture and staining of ApoE bound LNPs (C). The fluorescence of ApoE3 bound to LNPs that are captured by magnetic beads after incubation in human serum, heat treated human serum and recombinant ApoE3 in PBS, as determined by flow cytometry (D) (*n* = 2). Differences were considered statistically significant at *p* < 0.05 and were annotated as ns = non-significant, * = *p* ≤ 0.05, ** = *p* ≤ 0.01, *** = *p* ≤ 0.001 and **** = *p* ≤ 0.0001. The graphics in Figure 3C were generated with images provided by Servier Medical Art. Servier Medical Art by Servier is licensed under a Creative Commons Attribution 4.0 International License, https://creativecommons.org/licenses/by/4.0/

To investigate whether the thermal effects on ApoE are indeed the reason for the reduced uptake of LNPs, LNP uptake experiments were performed in the presence of HI ApoE with HMEC-1 cells. First, the effect of ApoE on LNP uptake by these cells was established by using ApoE3 in serum free media. A clear ApoE3 mediated LNP uptake was observed as BSA at similar concentrations did not facilitate the uptake of LNPs (Figure 3A). Interestingly, when ApoE3 was heat-inactivated before addition to serum free medium, uptake of LNPs by HMEC-1 was reduced by ≈50% (Figure 3B).

To directly demonstrate an effect of heat inactivation of serum on the binding of ApoE3 to LNPs, biotinylated LNPs were incubated in HI and NHI human serum. The LNPs were subsequently isolated via streptavidin decorated magnetic beads. Subsequently, ApoE was probed with a fluorescent antibody (Figure 3C). LNPs incubated with recombinant ApoE3 at a concentration similar to that of human serum was used as a positive control. Heat inactivation resulted in an almost fourfold reduction of fluorescence compared to untreated human serum, suggesting that heat treatment inhibits ApoE3 binding to the LNP surface (Figure 3D), most likely due to denaturing or aggregation of the protein. Alternatively, the reported conformational changes of ApoE3 after heat exposure that affect its binding to lipids [32], also affect its binding to LNPs. The significantly higher fluorescence of the positive control compared to untreated serum despite similar expected ApoE3 concentrations could be related to the absence of other proteins that can interfere with ApoE binding to the LNP surface.

Reportedly, not all LNP formulations are equally dependent on ApoE for their uptake by cells [37]. In fact, Miao et al. noted that uptake of mRNA LNPs containing ionizable lipid A6 was predominantly dependent on the ApoE3 concentration in the used medium, while the uptake of LNPs containing the ionizable lipids cKK-E12 or Syn-3 was mostly affected by albumin concentrations [38]. C12-200 (C12) is another ionizable lipid that is frequently used in preclinical LNP formulations, as it shows high transfection efficiency [39]. While the uptake of D-Lin-MC3-DMA LNPs is mostly driven by ApoE3-specific receptor mediated endocytosis [25], C12-200 LNP uptake seems to be mediated predominantly via ApoE-independent macropinocytosis [39]. It was therefore hypothesized that the uptake of C12 LNPs would be less affected by heat inactivation of FCS compared to MC3 LNPs.

**Figure 4:**
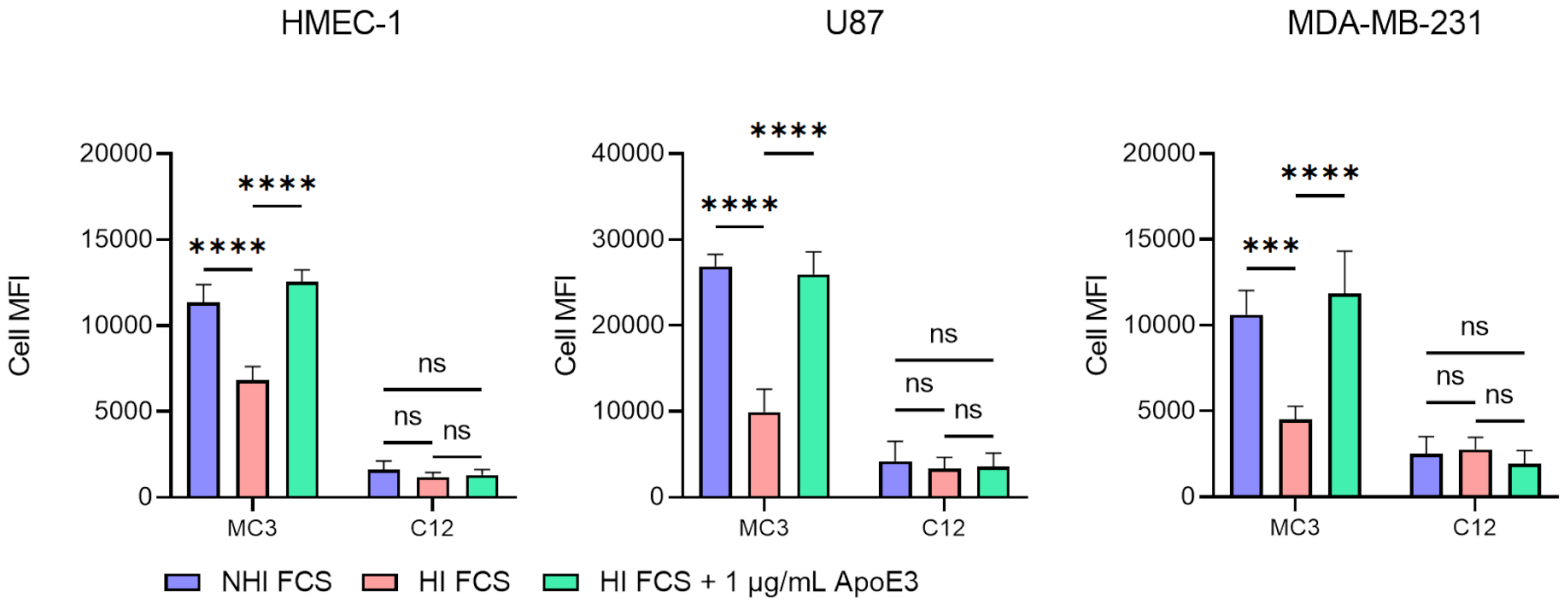
The uptake of MC3 LNPs is ApoE dependent and is affected by heat inactivation of FCS, while the uptake of C12 LNPs is not. Uptake of MC3 or C12 LNPs containing alexa fluor 647 labelled siRNA by HMEC-1, U87 and MDA-MB-231 in medium supplemented with NHI FCS, HI FCS or HI FCS with 1 µg/mL ApoE3. Analysed by flow cytometry (*n* = 3). Differences were considered statistically significant at *p* < 0.05 and were annotated as ns = non-significant, * = *p* ≤ 0.05, ** = *p* ≤ 0.01, *** = *p* ≤ 0.001 and **** = *p* ≤ 0.0001.

To verify this, we quantified uptake of C12 and MC3 LNPs by HMEC-1, U87 and MDA-MB-231 cells cultured in medium supplemented with HI or NHI FCS. Uptake by cells cultured in medium with HI FCS supplemented with 1 µg/mL recombinant ApoE3 was included as positive control. An overview of the physicochemical characteristics of the LNP formulations can be found in Supporting Information File 1 (Figure S1). Three cell lines were used to rule out cell type dependent differences in uptake of LNPs. Across all cell lines, use of HI significantly reduced the uptake of MC3 LNPs compared to NHI FCS. Addition of 1 µg/mL ApoE3 rescued the uptake in HI FCS to levels observed for NHI conditions. For C12 LNPs, HI had no effect on LNP uptake in HMEC-1, U87 or MDA-MB-231 cells. Moreover, no effect of ApoE3 supplementation was observed in any of the cell lines, indicating limited ApoE-dependent uptake of C12 LNPs (Figure 4).

To assess whether differences in uptake correspond to variations in RNA delivery efficiency, the two formulations were compared based on their ability to mediate luciferase knockdown in dual luciferase-expressing U87 and MDA-MB-231 cells. LNPs containing anti-firefly luciferase siRNA were incubated with the cells in HI FCS, NHI FCS or HI FCS supplemented with 1 µg/mL recombinant ApoE3.

**Figure 5:**
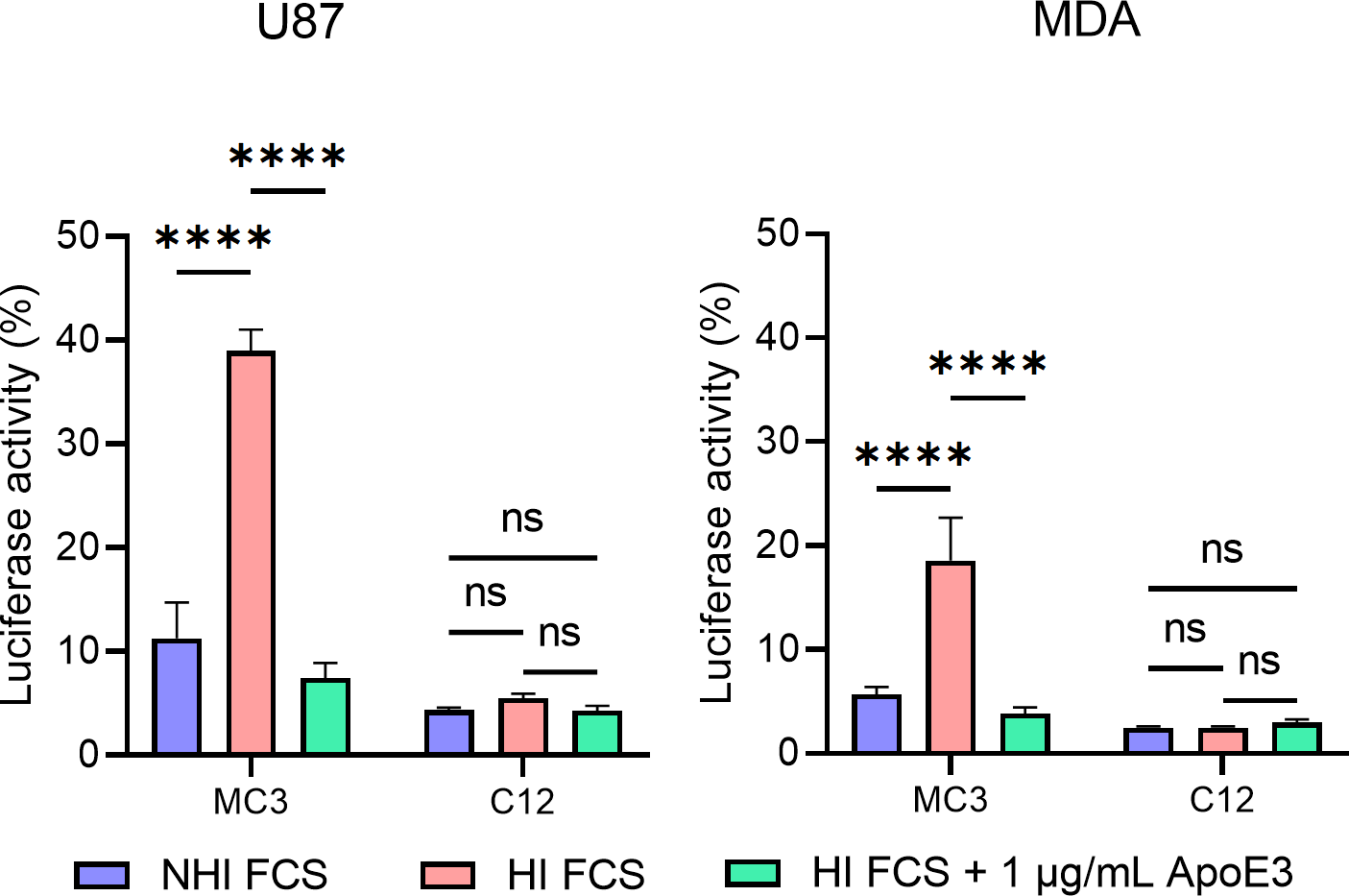
Heat inactivation of FCS and endogenous ApoE diminishes the firefly luciferase knockdown efficacy of MC3 LNPs, but not of C12 LNPs. Firefly luciferase activity in U87 and MDA-MB-231 48 h after uptake of siLuc loaded MC3 or C12 LNPs in medium supplemented with NHI FCS, HI FCS or HI FCS with 1 µg/mL ApoE3. Luciferase activity was analysed by measuring bioluminescence (*n* = 3). Differences were considered statistically significant at *p* < 0.05 and were annotated as ns = non-significant, * = *p* ≤ 0.05, ** = *p* ≤ 0.01, *** = *p* ≤ 0.001 and **** = *p* ≤ 0.0001.

In line with the uptake results, MC3 LNPs knocked down ≈89% of U87 and ≈94% of MDA-MB-231 firefly luciferase activity in NHI FCS, while the same particles in HI FCS achieved 61% and ≈81% silencing, respectively. The addition of recombinant ApoE improved knockdown efficiency to ≈93% for U87 and >95% for MDA-MB-231 (Figure 5).

Despite the fact that the C12 LNPs were applied at the same dose of 1 pmol siRNA per well, these LNPs were more efficient in knocking down luciferase activity than MC3 LNPs. This phenomenon has been reported previously [38]. In contrast to MC3 LNPs, the heat inactivation of FCS had no effect on the knockdown efficiency of C12 LNPs. Moreover, the addition of ApoE to HI FCS did not increase knockdown compared to HI FCS in U87 or MDA-MB-231 (Figure 5).

Collectively, our results show that the source and condition of serum components, such as ApoE, define the performance of MC3 LNPs in in vitro studies, while formulations containing other ionizable lipids are less affected.

## Conclusion

Heat treatment of FCS negatively affects uptake and transfection efficacy of D-lin-DMA-MC3 containing LNPs. This effect is caused by heat induced degradation/aggregation of ApoE. This implies that heat inactivation of FCS may alter the outcome of LNP delivery experiments. As such, in vitro LNP studies should be carefully designed to avoid such bias. The findings described here illustrate the importance of choosing the correct media when investigating LNP-cell interactions and emphasize that precise documentation of the methods and materials is crucial.

## Supporting Information

File 1 Physicochemical characteristics of MC3 and C12 LNPs.

## Data Availability

Data generated and analyzed during this study is available from the corresponding author upon reasonable request.
