# Supplementary material for: Serum heat inactivation diminishes ApoE-mediated uptake of D-Lin-MC3-DMA lipid nanoparticles
Source: Beilstein J Nanotechnol. 2025 May 30;16:740–8. doi: 10.3762/bjnano.16.57 (PMC12130627; doi:10.3762/bjnano.16.57)
Supplement: File 1 — Physicochemical characteristics of MC3 and C12 LNPs. [file Beilstein_J_Nanotechnol-16-740-s001.pdf]

BEILSTEIN JOURNAL OF NANOTECHNOLOGY

Supporting Information

for

Serum heat inactivation diminishes ApoE-mediated uptake of D-Lin-MC3-DMA lipid nanoparticles

Demian van Straten, Luuk van de Schepop, Rowan Frunt, Pieter Vader and Raymond M. Schiffelers

Beilstein J. Nanotechnol. **2025,** *16,* 740–748. doi:10.3762/bjnano.16.57

Physicochemical characteristics of MC3 and C12 LNPs

License and Terms: This is a supporting information file under the terms of the Creative Commons Attribution License (https://creativecommons.org/licenses/by/4.0). Please note that the reuse, redistribution and reproduction in particular requires that the author(s) and source are credited and that individual graphics may be subject to special legal provisions.
The license is subject to the *Beilstein Journal of Nanotechnology* terms and conditions: (https://www.beilstein-journals.org/bjnano/terms)

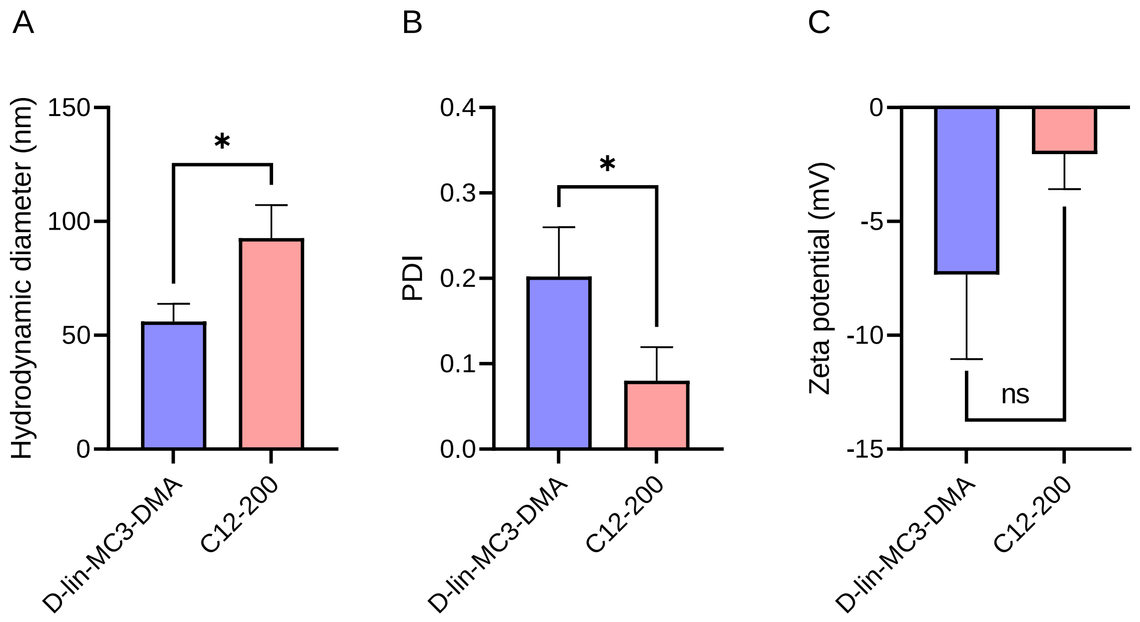

Figure S1: The physicochemical characteristics of MC3 and C12 LNPs. The hydrodynamic diameter of MC3 and C12 LNPs as determined by DLS (A). The polydispersity index (PDI) of MC3 and C12 LNPs as determined by DLS (B). The zeta potential of MC3 and C12 LNPs as determined by electrophoretic light scattering (C). Differences were considered statistically significant at $p < 0.05$ and were annotated as ns = non-significant, * = $p \leq 0.05$, ** = $p \leq 0.01$, *** = $p \leq 0.001$ and **** = $p \leq 0.0001$.
